# Holding vs Continuing GLP-1/GIP Agonists Before Upper Endoscopy: The OCULUS Randomized Clinical Trial

**DOI:** 10.1001/jamainternmed.2026.0027

**Published:** 2026-03-16

**Authors:** Akram I. Ahmad, Samita Garg, Jeffrey Jacobs, Zaid Ansari, Tasneem Jamal Al-Din, Ashraf Almomani, Sara Valencia, John Vargo, Arjun Chatterjee, Hassan Siddiki, Liang Hong, Michael A. Nicolas, Alaina Miller, Tilak Shah

**Affiliations:** 1 Department of Gastroenterology, Cleveland Clinic Florida, Weston; 2 Department of Gastroenterology, Cleveland Clinic, Cleveland, Ohio; 3 Department of Anesthesiology, Cleveland Clinic Florida, Weston; 4 Department of Gastroenterology, University of Missouri, Columbia; 5 Department of Medicine, MetroHealth Medical System, Cleveland, Ohio; 6 Department of Clinical Research, Cleveland Clinic Florida, Weston

## Abstract

**Importance:**

Glucagon-like peptide-1 (GLP-1) and glucose-dependent insulinotropic polypeptide (GIP) are known to increase the risk of retained gastric contents. High-quality data are lacking to guide periprocedure management of GLP-1 and GIP agonists.

**Objective:**

To compare the risk of clinically significant residual gastric volume (RGV) in patients who continue vs hold 1 dose of weekly or daily GLP-1 and GIP agonists prior to sedation.

**Design, Setting, and Participants:**

This randomized, single-masked clinical trial conducted at 2 large tertiary referral centers in the US included patients undergoing elective upper endoscopy (EGD) who were receiving GLP-1 or GLP-1/GIP agonists between July 2024 and May 2025. Eligible participants were adults aged 18 years or older, scheduled for EGD with or without colonoscopy, under moderate sedation or monitored anesthesia care, and taking a stable dose of a GLP-1 or GLP-1/GIP agonist for at least 1 month. Exclusion criteria were prior foregut surgery, achalasia, documented gastroparesis, RGV on previous endoscopy, gastric outlet obstruction, planned general anesthesia, or recent opioid use. Data were analyzed May 2025.

**Intervention:**

Participants were randomized to either continue their medication or hold 1 dose prior to the procedure.

**Main Outcomes and Measures:**

Clinically significant RGV, a composite of gastric contents that (1) precludes endoscopic examination, (2) requires premature termination or endotracheal intubation, and/or (3) results in an aspiration event that necessitates extended observation or monitoring, unplanned therapeutics, or hospital admission.

**Results:**

There were 60 patients (32 holding 1 dose, 28 continuing medication) in the preplanned interim analysis (median [IQR] age, 62.5 [55.5-67.5] years; 30 female [50.0%]). Clinically significant RGV occurred in 3.1% in the hold group vs 25.0% in the continue group (absolute difference, 21.9% [90% CI, 7.0%-36.7%]; *P* = .003). The trial was terminated early as risk exceeded the preestablished O’Brien-Fleming stopping boundary. In the EGD-only subgroup (35 patients), clinically significant RGV occurred in 46.7% in the continue vs 5.0% in the hold groups (absolute difference, 41.7% [90% CI, 17.9%-65.4%]; *P* = .001). In the EGD plus colonoscopy subgroup (25 patients), who were on clear liquids the day prior, no patients had clinically significant RGV.

**Conclusions and Relevance:**

This randomized clinical trial found that continuing GLP-1 or GIP agonist in the preprocedural period increased clinically significant RGV but did not increase the risk of other adverse events. Clear liquids the day prior to the procedure may mitigate the risk of clinically significant RGV regardless of GLP-1/GIP use.

**Trial Registration:**

ClinicalTrials.gov Identifier: NCT06533527

## Key Points

**Question:**

Does the use of glucagon-like peptide-1 (GLP-1) or glucose-dependent insulinotropic polypeptide (GIP) agonists have clinically significant effects on residual gastric volume (RGV) in the periprocedural period?

**Findings:**

In this randomized clinical trial including 60 US adults with a prespecified interim analysis, the rate of clinically significant RGV was 25.0% vs 3.1% when GLP-1/GIP agonists were continued vs held, respectively, in the periprocedural period. The difference was statistically significant and met the preestablished stopping boundary for study termination.

**Meaning:**

These results suggest that GLP-1 and GIP agonists treatment increases clinically significant RGV during the periprocedural period; however, there was no concomitant increase in aspiration-related adverse events.

## Teaser

This randomized clinical trial of US adults treated with stable doses of glucagon-like peptide-1 and/or glucose-dependent insulinotropic polypeptide agonists examines the effects on residual gastric volume.

## Introduction

Glucagon-like peptide-1 (GLP-1) receptor and glucose-dependent insulinotropic polypeptide (GIP) agonists were initially approved for the treatment of type 2 diabetes. Their use has exponentially increased after phase 3 trials demonstrated their effectiveness at inducing weight loss and glucose control.^1,2,3^ Their clinical use is expected to continue to increase, given associated improvement in outcomes with glucose control, cardiovascular disease (CVD), chronic kidney disease (CKD), metabolic dysfunction-associated steatohepatitis (MASH),^4^ and obstructive sleep apnea (OSA).

One of the potential explanations for the effectiveness of GLP-1 and GIP agonists at reducing postprandial hyperglycemia and inducing satiety is delayed gastric emptying.^5^ Possible mechanisms include inhibition of acetylcholine release via vagal pathways, increased production of nitric oxide, and modulation of GABAergic pathways in the central nervous system, all of which can influence vagal activity.^6^

Based on anecdotal reports and limited retrospective data that GLP-1 agonists could increase the risk of regurgitation and pulmonary aspiration of gastric contents during general anesthesia and moderate to deep sedation,^7,8^ the American Society of Anesthesiologists (ASA) issued a consensus statement in 2022. They recommended that patients receiving daily dosing hold GLP-1 agonists on the day of the procedure and patients receiving weekly dosing hold the drug a week before the procedure, regardless of the indication, dose, or type of procedure.^9^ Despite limited evidence, these recommendations were rapidly incorporated nationwide into routine practice for all sedated procedures (eg, endoscopy and cardiac catheterization) and operating rooms.

Given the scarcity of high-quality evidence and potential negative impact on delaying or cancelling necessary procedures, the American Gastroenterological Association (AGA) suggested a more individualized approach.^10^ Multisociety guidelines were subsequently published that emphasized the need for high-quality data.^11^ To date, no prospective randomized trials have addressed this issue. An upper endoscopy is the most accurate method to assess residual gastric volume (RGV), as the stomach is directly visualized during sedation. Our study aimed to evaluate the clinical impact of continuing vs holding GLP-1 and GIP agonists prior to upper endoscopy. We hypothesized that continuing these medications would be noninferior to holding 1 dose prior to the procedure with respect to clinically significant RGV.

## Methods

### Study Design

This randomized, single-masked, noninferiority clinical trial was conducted at 2 tertiary care centers in the Cleveland Clinic Enterprise (Cleveland Clinic in Weston, Florida, and Cleveland, Ohio) (Supplement 1). Both endoscopists and anesthesiologists were masked to the patients’ group assignments. The study was approved by the Cleveland Clinic Enterprise institutional review board and was registered on ClinicalTrials.gov (NCT06533527). All study participants provided written informed consent. This trial followed the Consolidated Standards of Reporting Trials (CONSORT) reporting guideline.

### Participants

Eligible participants were adults aged 18 years or older who were scheduled to undergo an elective upper endoscopic procedure under monitored anesthesia care or moderate sedation. Permitted procedures included esophagogastroduodenoscopy (EGD), endoscopic ultrasonography (EUS), or endoscopic retrograde cholangiopancreatography (ERCP). Patients undergoing elective colonoscopy were permitted to participate if they were concurrently undergoing an upper endoscopic procedure. Patients were eligible if they were taking a stable dose of a GLP-1 or GLP-1/GIP agonist for at least 1 month. Exclusion criteria were as follows: previous foregut surgery, achalasia, documented gastroparesis based on 4-hour solid phase gastric emptying study, previously reported retained gastric contents during upper endoscopy, known gastric outlet obstruction, planned general anesthesia, or chronic opioid use. Participants were recruited between July 2024 and May 2025.

### Interventions

The study participants were randomized into 2 groups. In the intervention group (referred to as the continue group): patients continued their medication at the same dosage and scheduled times. Patients in the control group (or hold group) were instructed to hold 1 dose of their medication, ie, a medication-free period of 7 to 13 days before upper endoscopy for weekly medications or at least 1 day for daily medications.

All patients followed the usual fasting instructions prior to anesthesia. Those scheduled for upper endoscopy only were maintained on a regular diet the day before (stopping at midnight) and clear liquids up to 2 hours prior to the procedure, whereas those who also had a colonoscopy and upper endoscopy were placed on a clear liquid diet 24 hours before the procedure.

### Main Outcomes

The primary outcome was clinically significant RGV in the 2 groups. Clinically significant RGV is a composite measure of RGV that either precludes endoscopic examination, requires premature termination of the procedure or endotracheal intubation, or results in an aspiration event that necessitates extended monitoring, unplanned therapeutics, or hospital admission.

A secondary outcome was increased RGV (RGV), a measure of any amount of solid content or more than 0.8 mL/kg of fluid content. To measure residual fluid content, endoscopists were instructed to aspirate all fluid from the stomach before examination or lavage. The amount of aspirated fluid was measured in a suction canister. During the trial, RGV did not require procedure termination, intubation, or result in adverse events.

### Sample Size

Based on previously published studies, we estimated a 0.5% and 7% rate of clinically significant RGV in nonusers of GLP-1 and GIP agonists vs users who do not hold the medications prior to the procedure, respectively. We calculated a sample size of 120 patients needed (60 in each group) to be 80% certain that the upper limit of a 1-sided 95% CI will exclude a difference in favor of the standard group of more than 3% (the noninferiority limit) using Farrington-Manning score test.

### Randomization and Masking

We used simple randomization using the REDCap randomization tool. To ensure concealed allocation, a computer-generated randomization sequence was uploaded by a statistician. A study coordinator (M.N. and A.M.) managed the randomization process and communicated the intervention to the patients. The investigators, endoscopists, anesthesiologists, and endoscopy nurses remained masked to the patients’ group assignments. To maintain masking, several measures were used. Prior to study initiation, we held meetings to discuss the study with the endoscopy nurses, the anesthesia department, and the gastroenterology department. We labeled electronic medical records and procedure boards of study participants to alert members of the clinical team (anesthesia, nursing, and gastroenterologists) that the patient was enrolled in the study. Finally, we informed the specific members who were anticipated to be involved with the procedure in advance about a study patient on their schedule.

### Interim Analysis

The study protocol prespecified interim analyses after enrollment of 20% and 50% of the target sample size. The interim analysis at 20% enrollment was conducted to reassess the adequacy of the sample size, given the lack of high-quality prospective data available at the time of study design. The interim analysis at 50% enrollment was planned to evaluate the primary outcome and to assess safety and futility. An O’Brien-Fleming group-sequential stopping boundary was applied (critical value = 2.5491), with a conditional power threshold of 20% used to guide futility assessment.

### Statistical Analysis

Data were summarized as mean (with SDs) or median (with IQRs) measures for continuous variables, and as counts with percentages for categorical variables. Statistical analyses were performed using SAS software, version 9.4 (SAS Institute Inc). The Farrington-Manning (score) noninferiority test was used for the primary outcome, conducted in both the intention-to-treat (ITT) and per-protocol (PP) fashion. The primary outcome was calculated based on the ITT analysis. A prespecified subgroup analysis for the primary outcome was conducted for patients who underwent upper endoscopy with colonoscopy (clear liquid diet the day prior to the procedure) or upper endoscopy only (regular diet the day prior to the procedure). Categorical variables were compared using Fisher exact test or the χ^2^ test, as appropriate, and continuous variables were analyzed using the Wilcoxon rank-sum test. Univariate exact logistic regression was performed; multivariable analysis was not conducted due to the limited sample size. An O’Brien-Fleming stopping boundary (critical value = 2.5491) was applied for the interim analysis. Statistical significance was set at a 1-sided *P* value of <.05.

## Results

**Figure 1.**
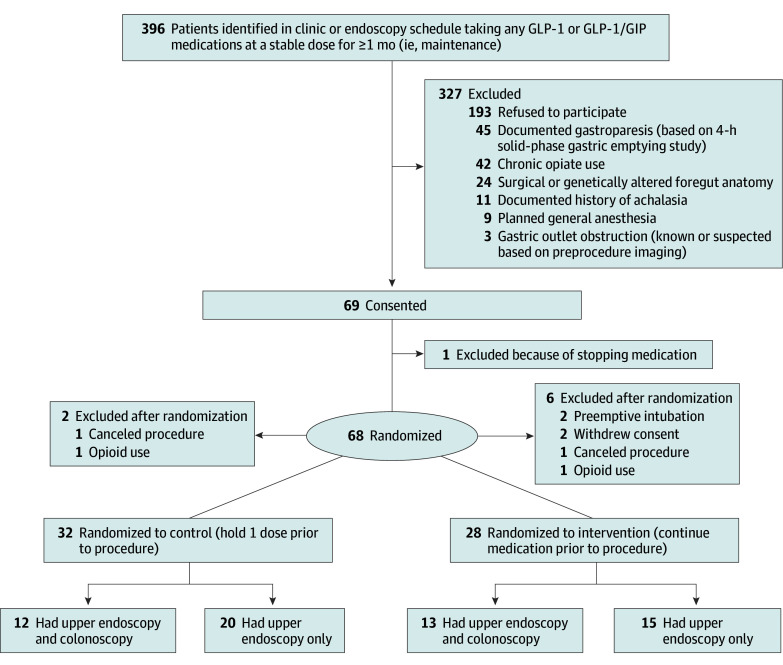
Flow Diagram of Participant Screening and Randomization GIP indicates glucose-dependent insulinotropic polypeptide; GLP-1, glucagon-like peptide-1.

Between July 2024 and May 2025, 396 candidates were identified and invited to participate in the study. Of these, 69 patients provided informed consent, and 68 were subsequently randomized (Figure 1). Thirty-two patients were assigned to the control (or hold) group (median [IQR] age, 63 [39-78] years; 15 female [47%]), including 20 who underwent upper endoscopy alone and 12 who underwent both upper endoscopy and colonoscopy. Twenty-eight patients were assigned to the intervention (continue) group, with 15 undergoing upper endoscopies alone and 13 undergoing both upper endoscopy and colonoscopy (Figure 1). Eight patients were excluded after randomization (Figure 1; eTables 1 and 2 in the Supplement 2). Baseline characteristics, procedure indications, and preprocedure symptoms did not differ between the 2 groups (Table).

**Table.** Baseline Characteristics of Participants by Study Group

Variable	Patients, No. (%)		*P* value
	Hold (n = 32)^a^	Continue (n = 28)^a^	
Age, median (range), y	63 (39-78)	63 (30-87)	.82
Sex			
Female	15 (47)	15 (55)	.60
Male	17 (53)	13 (45)	
Diabetes	10 (31)	7 (25)	.59
Hemoglobin A_1c_ >7%	6 (21)	7 (26)	.64
Hemoglobin A_1c_, median (range), %	6.4 (4.8-9.2)	6.4 (5.0-10.6)	.44
Body mass index, median (range)^b^	29.7 (23.6-49.9)	31.0 (20.2-40.9)	.57
Drug type			
Dulaglutide	4 (13)	3 (11)	.06
Semaglutide	18 (56)	9 (32)	
Semaglutide (oral)	2 (6)	1 (3)	
Tirzepatide	8 (25)	15 (54)	
Duration of GLP-1 or GLP-1/GIP agonist use, median (range), mo	6 (1-48)	6 (1-70)	.76
GLP-1 or GLP-1/GIP monotherapy	12 (38)	8 (29)	.46
Procedure indication			
Heartburn	5 (16)	3 (11)	.71
Dyspepsia	2 (6)	3 (11)	.66
Anemia	0	1 (4)	.47
Disease follow up	12 (38)	8 (29)	.46
Others	13 (41)	10 (36)	.70
Preprocedural symptoms			
Nausea	0	1 (4)	.47
Vomiting	0	1 (4)	.47
Heartburn	0	1 (4)	.47
Dyspepsia	1 (3)	1 (4)	>.99
Bloating	1 (3)	2 (7)	.59
Other symptoms	12 (38)	9 (32)	.66

Abbreviations: GIP, glucose-dependent insulinotropic polypeptide; GLP-1, glucagon-like peptide-1.

SI conversion factor: To convert hemoglobin A_1c_ to proportion of total hemoglobin, multiply by 0.01.

^a^ In the hold group, patients were instructed to hold 1 dose of their medication, for a medication-free period of 7 to 13 days before upper endoscopy; in the continue group, patients continued their medication at the same dosage and scheduled times.

^b^ Calculated as weight in kilograms divided by height in meters squared

At the 20% interim analysis (22 patients), only 1 patient in the continue group had clinically significant RGV, and no patient in the hold group had clinically significant RGV. Based on a revised assumption that the risk of clinically significant RGV was 9.0% in the continue group and 0% in the hold group, the recalculated sample size of 116 patients remained close to the original estimate, so no adjustments to the original sample size were recommended.

At the 50% interim analysis (60 patients), in the ITT analysis the rate of clinically significant RGV was 25.0% in the continue group and 3.1% in the hold group (absolute difference, 21.9% [90% CI, 7.0%-36.7%]; *P* = .003). The z-value of 2.75 exceeded the prespecified O’Brien-Fleming stopping boundary of 2.5491 (ie, *P* value of .0029 was less than O’Brien-Fleming α of .0054). All cases of clinically significant RGV occurred in the upper endoscopy-only subgroup (ie, no cases in the subgroup of patients who consumed a clear liquid diet and bowel preparation prior to the procedure). In the upper endoscopy only subgroup, the differences were even more pronounced. The rate of clinically significant RGV was 46.7% vs 5.0% in the ITT analysis of the continue vs hold groups, respectively (absolute difference, 41.7% [90% CI, 17.9%-65.4%]; *P* = .001). In this subgroup, the z-value of 3.09 markedly exceeded the prespecified O’Brien-Fleming stopping boundary of 2.5491 (ie, *P* value of .001 was less than O’Brien-Fleming α of .0054). Of note, all cases of clinically significant RGV were RGV that precluded adequate examination. There were no unanticipated cases of endotracheal intubation, aspiration, extended monitoring, or hospitalization. Because the z-value in the overall cohort for the primary outcome exceeded the prespecified stopping boundary, the study was terminated early.

**Figure 2.**
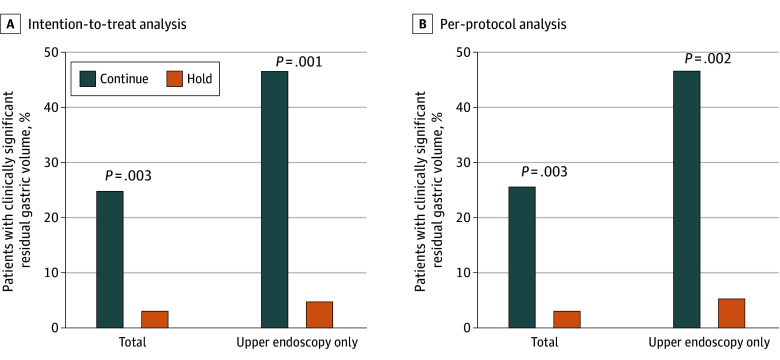
Bar Chart of Primary Outcome by Randomization Group in Intention-to-Treat and Per-Protocol Analyses In the intervention or hold group, patients were instructed to hold 1 dose of their medication, for a medication-free period of 7 to 13 days before upper endoscopy; in the control group, patients continued their medication at the same dosage and scheduled times.

PP analysis did not affect the primary outcome. Clinically significant RGV rates in the PP analysis were 25.9% and 3.2% in the continue and hold groups respectively (absolute difference, 22.7% [90% CI, 7.4%-38.0%]; *P* = .003). In the upper endoscopy-only subgroup, rates were 46.7% for the continue group vs 5.6% for the hold group (absolute difference, 41.1% [90% CI, 16.3%-66.0%]; *P* = .002) (Figure 2).

In the subgroup of patients who underwent both upper endoscopy and colonoscopy (ie, clear liquid diet on the day prior to the procedure), there were no clinically significant RGV events (ie, no events in hold or continue groups). The bowel preparation was deemed adequate in all patients who underwent colonoscopy (25 patients).

The secondary outcome of RGV without procedure termination, intubation, or adverse events did not occur in any patient who did not have the primary outcome of clinically significant RGV. The total volume of liquid suctioned from the stomach among patients who did not meet the primary outcome was similar between groups (mean [SD], 11 [12] mL in the control cohort vs 10 [16] mL in the intervention group; *P* = .30).

On univariate regression analysis, not undergoing colonoscopy (ie, solid diet on the day prior to the procedure) was significantly associated with increased risk of clinically significant RGV (OR, 9.66 [95% CI, 1.82-∞]; *P* = .009). GLP-1/GIP drug type and hemoglobin A_1c_ (HbA_1c_) above 7% were not associated with the primary outcome. Among patients on weekly GLP-1/GIP medications, the proportion of patients without clinically significant RGV was significantly higher in those who held the medication for more than 3 days (38 patients [92.7%]) compared with 3 days or less (5 patients [62.5%]) (*P* = .047).

Only 1 patient reported nausea and vomiting, and there was no clinically significant RGV or increased RGV in this individual. Other symptoms (eg, heartburn, dyspepsia) were not associated with the primary or secondary outcome. In fact, none of patients with clinically significant RGV in either group reported any upper gastrointestinal (GI) symptoms on the day of the procedure. The 1 patient in the hold group with clinically significant RGV was on daily oral semaglutide.

## Discussion

Current guidelines and consensus statements on the management of GLP-1 and GIP in the perioperative period are based on retrospective observational studies.^12,13,14^ To our knowledge, this is the first randomized clinical trial to assess the impact of continuing vs holding GLP-1 and GIP receptor agonists on clinically significant RGV in the preoperative period. Contrary to our hypothesis, there was a marked increase in the risk of clinically significant RGV in patients who did not hold a dose of the medication prior to the procedure. All cases of clinically significant RGV were RGV that precluded endoscopic examination. There were no cases of unplanned endotracheal intubation, aspiration, or hospitalizations due to RGV. Although anesthesia-related adverse events were not observed in our cohort, the study was not powered to detect differences in uncommon but clinically important safety outcomes. The absence of observed events should not be interpreted as evidence of safety for administering anesthesia with an unprotected airway in a population in which a substantial proportion of patients may present with a full stomach. In exploratory subgroup analyses, a clear liquid diet on the day prior to the procedure appeared to eliminate this risk. Also, upper GI symptoms such as nausea, vomiting, and dyspepsia were not associated with clinically significant RGV changes. In fact, none of patients with clinically significant RGV had any upper GI symptoms. While not a specific aim of this study, screening patients on these medications for symptoms is often a decisionpoint in periprocedural management, and therefore, this finding should be taken into consideration when creating future care paths.

Our results are most relevant to patients undergoing foregut surgery or sedated procedures without general anesthesia (eg, endoscopy, cardiac catheterization, and interventional radiology procedures). In the setting of upper endoscopy, our study suggests a significantly higher risk of clinically significant RGV if the patient follows standard fasting guidelines (solid food permitted until 8 hours prior to the procedure) and does not withhold a dose of the GLP-1/GIP medication prior to the procedure. Patients undergoing upper endoscopy often have gastrointestinal conditions that impact gastric motility, so it is possible the magnitude of risk observed in our trial may not extrapolate to other procedures.

A clear liquid diet on the day prior to the procedure may eliminate this risk, although we would emphasize that this finding was based on a subgroup analysis and that the study was not specifically powered to assess the impact of a clear liquid diet. That said, our findings are consistent with the existing literature. In a 2025 meta-analysis of 23 observational studies consisting of 262 018 patients, a clear liquid diet on the day prior to the procedure significantly reduced the risk of RGV compared with standard fasting (OR, 0.28; 95% CI, 0.22-0.36; *P* < .001; *I^2^* = 0%).^15^ In our subgroup analysis, continuation of GLP-1 and/or GIP agonists in conjunction with a clear liquid diet did not appear to increase residual gastric volume, suggesting that medication discontinuation may not be necessary when this dietary strategy is employed. However, further adequately powered studies are needed to directly compare this approach with medication withholding. In addition, patient preferences and potential consequences of medication interruption—particularly effects on glycemic control among patients receiving monotherapy—should be carefully considered.

In some studies, GLP-1 and GIP were associated with a higher incidence of inadequate bowel preparation,^16,17,18,19^ which could be a separate reason to consider holding a dose of these medications prior to colonoscopy. All patients undergoing colonoscopy in our study had an adequate bowel preparation. The impact of these medications on bowel preparation adequacy for colonoscopy is being assessed in an ongoing randomized trial (OCULUS 2). Unlike prior observational studies, this randomized trial assigned patients to either hold or continue GLP-1 and GIP agonists, thereby minimizing the risk that observed differences in outcomes were due to confounding rather than the intervention itself. To further reduce bias, all staff were masked to group assignments, mitigating the risk of detection bias. The study was conducted at 2 centers with distinct patient populations, enhancing the generalizability of our findings. Although both sites are tertiary referral centers, the Cleveland site cares for a substantial proportion of patients covered by Medicaid or without insurance, whereas the South Florida site serves a primarily insured population with a high proportion of Hispanic patients (approximately 30%).

### Limitations

Our study had limitations: we elected to include patients undergoing combined upper endoscopy and colonoscopy (ie, on a clear liquid diet), as we were particularly interested in outcomes in this subgroup. While this decision could have affected the ITT analysis if 1 group included disproportionately more of these patients, the randomization process yielded a nearly equal distribution across groups. To minimize confounding, we excluded patients with risk factors for gastroparesis (including opiates) other than diabetes, which may limit generalizability to these populations. Because inadequate glycemic control is associated with delayed gastric emptying, we prespecified that we would monitor—but not control for—HbA_1c_, unless interim analyses revealed a significant imbalance. At the 50% enrollment analysis, the proportion of patients with HbA_1c_ above 7% did not differ between groups. To minimize variability in gastric motility effects, we restricted inclusion to patients receiving a stable dose of therapy for at least 1 month and excluded those undergoing dose escalation. Finally, the study was not powered to detect differences among individual GLP-1 and GIP medications (eg, short acting vs long acting or varying doses) or to evaluate the impact of medication withholding as a continuous variable. While these factors limit the scope of our conclusions, they also identify key questions that warrant further study. We did not evaluate the glucose control in this study as there was only 1 dose of GLP-1 and/or GIP withheld. The other diabetes medication regimen was not changed in this study and there were few patients on insulin which was adjusted accordingly prior to the procedure. It is important to note that baseline HbA_1c_ was 6.4% in both groups which is below the American Diabetes Association (ADA) target for good glycemic control.

## Conclusions

In conclusion, continuation of GLP-1 and/or GIP agonist therapy in the preprocedural setting was associated with a significant increase in residual gastric volume. Notably, patients who met the primary outcome were predominantly asymptomatic, indicating that symptom-based strategies may be insufficient for periprocedural risk stratification.

## Notes

Supplement 1. Trial Protocol

Supplement 2. eTable 1. Excluded from intention to treat analysis after randomization eTable 2. Excluded from per-protocol analysis

Supplement 3. Data Sharing Statement
