# Supplementary material for: Holding vs Continuing GLP-1/GIP Agonists Before Upper Endoscopy: The OCULUS Randomized Clinical Trial
Source: JAMA Intern Med. 2026 Mar 16;186(5):578–84. doi: 10.1001/jamainternmed.2026.0027 (PMC12993733; doi:10.1001/jamainternmed.2026.0027)
Supplement: Supplement 1. — Trial Protocol [file jamainternmed-e260027-s001.pdf]

Randomized trial of holding vs. continuing incretin-based therapies before upper endoscopy

Corresponding author:
Akram I Ahamd
Email: ahmada5@ccf.org
Address: 2950 Cleveland Clinic Blvd, Weston, FL 33331

Principal Investigator:
Tilak Shah, M.D.
Email: shaht3@ccf.org
Address: 2950 Cleveland Clinic Blvd, Weston, FL 33331

Background:

Incretin-based therapies include GLP-1 receptor agonists (GLP-1RAs) and dual GLP1-RA and gastric inhibitory polypeptide (GIP) agonists. These drugs are commonly prescribed for diabetes and more recently, their use has risen exponentially as a primary therapy for weight loss. These medications can decelerate gastric emptying and diminish bowel motility by inhibiting peristalsis at the GLP-1 receptors situated in myenteric neurons. Therefore, there exists a theoretic potential for gastroparesis, retained gastric contents, perioperative regurgitation, and pulmonary aspiration syndrome, and this is a concern for anesthesiologists and patients taking these medications.

The American Society of Anesthesiologists recently released a consensus-based statement that recommends patients on daily dosing hold incretin-based therapy the day of the procedure, and those on weekly dosing hold the medication a week before the procedure. The evidence for these recommendations is based on retrospective studies and case reports, and therefore the conclusions may be overly proscriptive. Furthermore, holding incretin-based therapy poses a risk for worsening diabetes control and more importantly, could cause unnecessary delays in care if a patient's procedure is postponed because they failed to hold the medication based on the recent recommendations (1-3).

Study Objective:

To assess whether holding incretin-based therapy before endoscopy reduces the likelihood of clinically relevant Residual Gastric Volume (RGV).

Hypothesis:

Holding incretin-based therapy is non-inferior to continuing incretin-based therapy for reducing the risk of clinically relevant RGV.

Study design:

Randomized Non-Inferiority Controlled Trial.

Blinding:

Single-blind: endoscopist and anesthesia team will be blinded to study group assignments.

Primary Outcomes:

The primary outcome is clinically significant residual gastric volume defined as a composite of the following variables:

- Residual gastric volume that precludes adequate endoscopic examination
- Residual gastric volume that necessitates premature termination of the endoscopy procedure
- Need for endotracheal intubation due to stomach contents.
- Occurrence of aspiration events requiring extended observation/monitoring, unplanned therapeutics, and/or hospital admission

Secondary Outcomes:

- Presence of any solid food
- Presence of moderate liquid content
- Increased RGC defined as any amount of solid content or > 0.8 mL/Kg of fluid content (measured from the aspiration/suction canister).
- Aspiration events up to 48 hours after endoscopy
- Differences in primary and secondary outcomes between different medications

Inclusion Criteria:

- Patients using incretin-based therapies at a stable dose for more than 1 month.
- Understand the study procedures and will be able to provide written informed consent to participate in the study.
- Patients scheduled for outpatient esophagogastroduodenoscopy (EGD), combined EGD and colonoscopy, endoscopic ultrasound (EUS), or endoscopic retrograde cholangiopancreatography (ERCP) under monitored anesthesia care (MAC) or moderate sedation.

Exclusion Criteria:

- Documented history of gastroparesis (based on a4-hour solid-phase gastric emptying study)
- Known history of achalasia
- Surgical or genetically altered foregut anatomy
- Known gastric outlet obstruction or pre-procedure imaging suggestive of gastric outlet obstruction.
- Patients who did not follow the standard NPO (nil per oral) instructions.
- Patients who have planned endotracheal intubation (ETT) during their upper endoscopy procedure (EGD, EUS, or ERCP).
- Ongoing opiate use
- Unable or unwilling to give written consent
- Previous endoscopy with food retention (in the last 5 years or the last available EGD if none in 5 years)

Randomization:

- Patients will be randomly assigned to either continue incretin-based therapy as prescribed ("continue" group) or to hold therapy as outlined in the ASA guidance recommendations ("hold" group).
- Simple randomization will be applied post-enrollment, utilizing REDCap serial numbers with pre-determined interventions ("continue" vs. "hold"). The randomization table of allocation will be completed by a statistician on REDCap to ensure concealed allocation.

Study Operation:

- The candidates for the study will be determined either during the gastroenterology clinic visit OR based on review of records by a member of the research team of patients scheduled for upper endoscopy, EUS, and ERCP cases three to four weeks in advance
- Patients' invitation
    - For patients who are scheduled for the procedure through the gastroenterology clinic, the patient's clinic provider will invite the patient for the study during the office visit or with a phone call.
    - For patients who are not scheduled via the above route, a letter of invitation will be sent to the patient from the physician completing the procedure through the US mail or MyChart.
- Patient consent
    - Interested patients in the clinic setting will be given the option to complete a research encounter during the same day, including in-person consent if interested, or complete the process remotely over the phone and through DocuSign.
    - Patients who are not identified in clinic but through chart review will receive a phone call and phone encounters will be completed with interested potential participants and will include an explanation of the study and the consent process

(phone scripts attached). DocuSign will be used to send the study consent to the participants.

- After obtaining consent, the patient information will be entered into the REDCap which will give the patient a REDCap serial number.
- A randomization tool in the REDCap will determine the intervention (The randomization table of allocation will be completed by a statistician on REDCap to ensure concealed allocation).
- The patient will be informed about the intervention during the clinic visit by a study member (not the endoscopist) or over the phone.
- A few days before the intervention, the patient will receive a call as a reminder of the intervention and instruction from a study member (not the endoscopist).
- On the endoscopy day, the proceduralist and anesthesia team will be blinded to the patient's group assignment. To ensure that blinding is maintained, a sticker will be placed on the patient physical chart to remind the provider to avoid asking about the study intervention.
- A research coordinator/investigator will confirm intervention compliance and record the variables of interest for the study.
- Information will be directly entered into the Redcap data collection sheet or recorded on a physical paper-based collection sheet and transferred immediately to Redcap on the same day. The physical sheets will be shredded on the day of the procedure.
- A phone call to the patient within the first week after the procedure to follow up and exclude aspiration events. If patient cannot be contacted, then a member of the study team will review patient records to identify any emergency department visits or hospitalizations related to a post-procedure aspiration event.

Electronic Consent operation

- An investigator or research coordinator will contact the invited candidates over the phone 2-4 weeks before the scheduled procedure. The study purpose, intervention, adverse events, and consenting process will be discussed over the phone. The encounter will be documented in the patient chart as a phone encounter.
- Subjects interested in the study will be provided with consent through DocuSign with instructions to complete online consent signing.
- After all parties complete the consent, a randomization of the study intervention will be done through Redcap.
- A follow-up phone call will be made to communicate the instructions to the participants.

Rational for electronic consent (E-consent)

The study is not feasible with conventional consent, especially for open access procedures for the following reasons:

- The duration for participant involvement in the study is short, and the study intervention is only applied one week before the procedure, which would make it impractical to bring the patient for an extra encounter and would decrease the chance for enrollment.
- Many procedures are scheduled in an open-access fashion, which means that the patient is scheduled for the procedure by a provider other than the performing physician and/or from a different department.
- The intervention for the study must be completed one week before presenting the procedure, which requires that the patient be enrolled before the procedure date.

Sample Size Determination:

- Previous retrospective studies indicate that 5-7% of GLP-1RA users have increased RGV compared to less than 0.5% in non-users.
- If there is a significant difference in favor of the experimental ("hold") group of 7%, then 120 patients are needed (60 in each group) to be 80% certain that the upper limit of a one-sided 95% confidence interval (or equivalently a 90% two-sided confidence interval) will exclude a difference in favor of the standard group of more than 3% (the non-inferiority limit) using Farrington-Manning's score test.
- Because estimates from previous retrospective studies are for the outcome of residual gastric volume, they may not accurately reflect our primary outcome, which is a more pragmatic and clinically useful outcome. Therefore, we plan to conduct an interim analysis at 20% recruitment and may adjust sample size if necessary, based on point estimates derived from the interim analysis.

Study Variables

Date of birth, gender, race, body mass index (BMI), type of incretin-based therapy used, incretin therapy dose, pre-endoscopy clinical symptoms, presence of diabetes, last HBA1c if available, detailed endoscopic reports, gastric residual content on endoscopy, and RGC-related events as described above. Please refer to the data collection sheet for details of the variables above.

Study Statistics

The Statistics will be done with the help of a Clevland Clinic statistician. Data will be extracted in a deidentified form directly from Redcap for analysis.Once data has been collected, we will use STATA version 14.1 (STATA Corp, College Station, TX, USA) for statistical analysis. We will use statistics of central tendency (mean, median, and mode) for most values. Data normality will be tested using Kolmogorov–Smirnov test. We will use Pearson's chi-square tests for categorical variables and T-test for continuous variables. A P-value of < 0.05 will be considered significant for all statistical analyses. The primary outcome will be calculated using chi-square or Mann-Whitney test. For the primary outcome, an intention-to-treat analysis will be utilized. The prespecified subgroups of endoscopy and colonoscopy or endoscopy only will be analyzed with intention-to-treat analysis.

Interim Analysis and Data Monitoring Committee (DMC)

The study of DMC will include the following: The gastroenterology program director at Weston, a gastroenterologist, and a Cleveland Clinic statistician. Meetings will be held at 20%, 50, and 100% of recruitment to ensure data security compliance, appropriate rate of recruitment, and study adverse events monitoring. A report for each meeting will be sent to the IRB. The DMC will not be blinded for study groups. Interim analysis will be conducted after 20 and 50% of recruitment at the second DMC meeting, and the study will be stopped if inferiority is confirmed. O'Brien–Fleming stopping boundary was set as the stopping boundery for the study.

Data Security

Data, including patient information and consent, will be stored electronically in the Redcap system\DocuSign which is password-protected and on the secure Cleveland Clinic network. If physical paper sheets were used, the physical paper sheets' information would be transferred to Redcap and shredded on the day of data gathering.

Possible Harm to Subjects and Adverse Events

The study risk is related to continuing incretin-based therapy in the periprocedural time. Medication continuation has a theoretical risk of increased residual gastric content, although there is no strong evidence to support that. Increased gastric residual content can lead to prolonged monitoring time, increased procedure time, premature termination of the procedure, unexpected urgent endotracheal intubation, or aspiration events.

Breach of confidentiality is another potential harm, and it will be minimized by the data security plan described above. The study does not involve prescribing incretin-based therapy.

All serious unexpected adverse events will be recorded and reported to the IRB in a timely fashion. Adverse events (study outcome) will be reported to the IRB after each DMC meeting.

References

1- Kobori T, Onishi Y, Yoshida Y, et al. Association of glucagon-like peptide-1 receptor agonist treatment with gastric residue in an esophagogastroduodenoscopy. *J Diabetes Investig*. 2023;14(6):767-773. doi:10.1111/jdi.14005

2- Silveira SQ, da Silva LM, de Campos Vieira Abib A, et al. Relationship between perioperative semaglutide use and residual gastric content: A retrospective analysis of

patients undergoing elective upper endoscopy. *J Clin Anesth*. 2023;87:111091. doi:10.1016/j.jclinane.2023.111091

3- Joshi G, Abdelmalak B, Weigel W, et al. *American Society of Anesthesiologists Consensus-Based Guidance on Preoperative Management of Patients (Adults and Children) on Glucagon-Like Peptide-1 (GLP-1) Receptor Agonists*. Released June 29, 2023. Accessed from asahq.org on January 16, 2024.
