# Supplementary material for: Holding vs Continuing GLP-1/GIP Agonists Before Upper Endoscopy: The OCULUS Randomized Clinical Trial
Source: JAMA Intern Med. 2026 Mar 16;186(5):578–84. doi: 10.1001/jamainternmed.2026.0027 (PMC12993733; doi:10.1001/jamainternmed.2026.0027)
Supplement: Supplement 2. — eTable 1. Excluded from intention to treat analysis after randomization eTable 2. Excluded from per-protocol analysis [file jamainternmed-e260027-s002.pdf]

Supplemental Online Content

Ahmad AI, Garg S, Jacobs J, et al. Randomized clinical trial of holding vs continuing GLP-1/GIP1 agonists before upper endoscopy. *JAMA Intern Med.* Published online March 16, 2026. doi:10.1001/jamainternmed.2026.0027

eTable 1. Excluded from intention to treat analysis after randomization

eTable 2. Excluded from per-protocol analysis

This supplemental material has been provided by the authors to give readers additional information about their work.

© 2026 Ahmad AI et al. *JAMA Internal Medicine*.

eTable 1: Excluded from intention to treat analysis after randomization

**Reason for Exclusion After Randomization**	**Number of Patients**	**Group**
Withdrew consent before the procedure	2	2 (intervention)
Cancelled procedure	1	1 (Control)
Rescheduled the procedure with a plan for general anesthesia	1	1 (intervention)
Chronic opioid use was identified after randomization but before the procedure	2	1 (intervention), 1 (Control)
Preemptive endotracheal intubation	2*	2 (intervention)
**Total excluded**	8	6 (intervention), 1 (Control)

* Both patients were scheduled for a colonoscopy and upper endoscopy. Blinding was broken, and anesthesia proceeded with preemptive endotracheal intubation. Neither patient had solid gastric contents or increased residual gastric volume of fluid.

© 2026 Ahmad AI et al. *JAMA Internal Medicine*.

eTable 2: Excluded from Per-protocol analysis

**Reason for Exclusion After Randomization**	**Number of Patients**	**Group**
Chronic opioid use was identified after the procedure was completed.	1*	1 (intervention)
The patient consented for the procedure as an outpatient but was admitted before the scheduled procedure for abdominal pain. He therefore underwent the procedure in the inpatient setting	1	1 (Control)
**Total excluded**	2	1 (intervention), 1 (Control)

*The patient was scheduled for upper endoscopy and met the study's primary outcome of clinically significant residual gastric volume.

© 2026 Ahmad AI et al. *JAMA Internal Medicine*.
