# Supplementary material for: Holding vs Continuing GLP-1/GIP Agonists Before Upper Endoscopy: The OCULUS Randomized Clinical Trial
Source: JAMA Intern Med. 2026 Mar 16;186(5):578–84. doi: 10.1001/jamainternmed.2026.0027 (PMC12993733; doi:10.1001/jamainternmed.2026.0027)
Supplement: Supplement 3. — Data Sharing Statement [file jamainternmed-e260027-s003.pdf]

Data Sharing Statement

Ahmad. Holding vs Continuing GLP-1/GIP Agonists Before Upper Endoscopy. *JAMA Intern Med*. Published March 16, 2026. doi:10.1001/jamainternmed.2026.0027

Data
Additional Information: NCT06533527
Data available: No
